# CRP Regulates D-Lactate Oxidation in *Shewanella oneidensis* MR-1

**DOI:** 10.3389/fmicb.2017.00869

**Published:** 2017-05-16

**Authors:** Takuya Kasai, Atsushi Kouzuma, Kazuya Watanabe

**Affiliations:** School of Life Sciences, Tokyo University of Pharmacy and Life Sciences,Hachioji, Japan

**Keywords:** CRP, transcriptional regulation, lactate, *Shewanella*, catabolism

## Abstract

*Shewanella oneidensis* MR-1 is a heterotrophic facultative anaerobe that respires using various organic and inorganic compounds. This organism has served as a model to study bacterial metabolic and regulatory systems that facilitate their survival in redox-stratified environments. The expression of many anaerobic respiratory genes in MR-1, including those for the reduction of fumarate, dimethyl sulfoxide, and metal oxides, is regulated by cyclic AMP receptor protein (CRP). However, relatively little is known about how this organism regulates the expression of catabolic enzymes catalyzing the oxidation of organic compounds, including lactate. Here, we investigated transcriptional mechanisms for the *lldP* (SO_1522) and *dld* (SO_1521) genes, which encode putative lactate permease and D-lactate dehydrogenase, respectively, and demonstrate that CRP regulates their expression in MR-1. We found that a *crp*-deletion mutant of MR-1 (Δ*crp*) showed impaired growth on D-lactate. Complementary expression of *dld* in Δ*crp* restored the ability to grow on D-lactate, indicating that the deficient growth of Δ*crp* on D-lactate is attributable to decreased expression of *dld*. *In vivo* transcription and *in vitro* electrophoretic mobility shift assays reveal that CRP positively regulates the expression of the *lldP* and *dld* genes by directly binding to an upstream region of *lldP*. Taken together, these results indicate that CRP is a global transcriptional regulator that coordinately regulates the expression of catabolic and respiratory pathways in MR-1, including D-lactate dehydrogenase and anaerobic terminal reductases.

## Introduction

The genus *Shewanella* belongs to the class *Gammaproteobacteria* and is widely distributed in nature, including marine, freshwater, sedimentary, and soil environments (Venkateswaran et al., 1999; Hau and Gralnick, 2007; Fredrickson et al., 2008; Rodionov et al., 2011). Members of this genus are able to utilize a variety of electron acceptors for respiration, such as insoluble solid compounds (e.g., iron and manganese oxides) and soluble organic and inorganic compounds [e.g., oxygen, fumarate, nitrate, nitrite, dimethyl sulfoxide, and trimethylamine *N*-oxide (TMAO)] (Myers and Nealson, 1988; Fredrickson et al., 2008). The electron-acceptor versatility of this genus may have evolved to allow survival in redox-stratified environments, such as oxic/anoxic interfaces in sediments, where available electron acceptors frequently change.

*Shewanella oneidensis* MR-1 is the most extensively studied strain in the genus *Shewanella*. This organism has served as a model to study how bacteria adapt to redox-stratified environments (Fredrickson et al., 2008). Previous studies have shown that MR-1 expresses multiple anaerobic terminal reductase genes, including those for the reduction of metal oxides (*omcA* and *mtrCAB*),fumarate (*fccA*), and DMSO (*dmsEFAB*), when alternative electron acceptors, e.g., oxygen, are limited (Pirbadian et al., 2014; Barchinger et al., 2016). The simultaneous expression of these genes results in the formation of a dynamic respiratory electron-transfer network consisting of periplasmic and membrane-bound c-type cytochromes, enabling this strain to efficiently discharge electrons to various electron acceptors in response to changes in the environmental redox state (Sturm et al., 2015). Cyclic AMP (cAMP) receptor protein (CRP) plays a central role in the transcriptional regulation of these anaerobic respiratory genes (Saffarini et al., 2003; Fredrickson et al., 2008; Charania et al., 2009; Murphy et al., 2009; Dong et al., 2012; Kasai et al., 2015; Kouzuma et al., 2015). To cite an instance, CRP directly binds to the upstream regions of *omcA* and *mtrC* in the presence of cAMP, and activates the transcription of these metal-reduction genes (Kasai et al., 2015). Recent studies have also shown that CRP and cAMP are involved in the regulation of aerobic respiration in MR-1 (Fu et al., 2013; Zhou et al., 2013; Gao et al., 2015; Jin et al., 2016; Yin et al., 2016). These findings are intriguing, since our knowledge of CRP derives mostly from studies on *Escherichia coli* and other enterobacteria in which CRP is shown to regulate carbon catabolite repression by glucose (Botsford and Harman, 1992; Kolb et al., 1993). Further studies to identify the physiological functions of the cAMP/CRP regulatory system in *Shewanella* are therefore needed.

In contrast to the relatively well-investigated regulatory mechanisms for the respiratory genes, less is known about how MR-1 regulates catabolic pathways that donate electrons to respiratory pathways. MR-1 preferably utilizes low-molecular-weight organic acids, particularly lactate, as carbon and energy sources under aerobic and anaerobic conditions (Scott and Nealson, 1994; Serres and Riley, 2006). A previous study has identified respiratory L- and D-lactate dehydrogenase (LDH) genes as responsible for the selective oxidation of these isomers to pyruvate in MR-1 (Pinchuk et al., 2009). In this strain, L-LDH is comprised of three subunits encoded by the *lldEFG* genes (SO_1520 to SO_1518), whereas D-LDH is encoded by the *dld* gene (SO_1521), a distant homolog of a FAD-dependent LDH gene in yeast (Pinchuk et al., 2009). A previous study has also demonstrated that LlpR (L-lactate-positive regulator, SO_3460) is required for L-lactate utilization by MR-1, suggesting that this regulator is involved in the transcriptional activation of *lldEFG* (Brutinel and Gralnick, 2012). This work has also uncovered that MR-1 preferentially utilizes D-lactate when both L- and D-lactate isomers are present (Brutinel and Gralnick, 2012). In addition, the expression of *dld* is up-regulated under oxygen-limited conditions (Barchinger et al., 2016) and high electrode potential-applied conditions in bioelectrochemical systems (Nakagawa et al., 2015), suggesting the possibility that the ability of MR-1 to utilize D-lactate is affected by electron acceptors. These observations suggest that D-lactate is an important catabolic substrate for *Shewanella* spp., particularly when they grow in anaerobic environments. Nevertheless, the molecular mechanisms underlying the regulation of D-LDH in this genus remain to be elucidated.

Here, we examined the involvement of CRP in the regulation of D-lactate oxidation in *S. oneidensis* MR-1. We hypothesized that, to thrive in nutrient-limited conditions, bacteria should coordinately regulate electron-donating catabolic pathways (e.g., D-LDH) and electron-consuming respiratory pathways (e.g., metal reductases), and that CRP is involved in this regulation. Findings presented herein provide insights into the coordinated regulation of catabolic and respiratory pathways in bacteria that thrive in the natural environment.

## Materials and Methods

### Chemicals

Chemicals used in this study were of the highest commercially available purity and purchased from Kanto Chemical (Tokyo, Japan), Wako Pure Chemical (Tokyo, Japan), and Tokyo Kasei Kogyo (Tokyo, Japan). The stock solution of D-lactic acid was neutralized to pH 7.4 with sodium hydroxide before use as a growth substrate for *S. oneidensis* strains.

### Bacterial Strains, Plasmids, and Growth Condition

Bacterial strains and plasmids used in the present study are listed in **Table 1**. *Escherichia coli* strains were cultivated in Luria-Bertani (LB) or 2 × yeast extract-tryptone (2 × YT) medium at 37°C. The *E. coli* mating strain (WM6026) required 100 μg/ml 2,6-diaminopimelic acid (DAP) for growth. *S. oneidensis* strains were cultured at 30°C in LB or minimal medium (MM) (Nakagawa et al., 2015) containing a racemic mixture of DL-lactate, D-lactate, L-lactate, or pyruvate as the carbon and energy source. *S. oneidensis* strains were grown under aerobic or anaerobic TMAO-reducing conditions, since a CRP-deletion mutant (Δ*crp*) cannot utilize other electron acceptors (Saffarini et al., 2003). For aerobic cultivation, MM supplemented with each substrate (10 mM) in a test tube was inoculated with an *S. oneidensis* strain and shaken at 180 rpm. For anaerobic cultivation, MM supplemented with each substrate and TMAO (10 mM or 30 mM) in a test tube was inoculated with an *S. oneidensis* strain and incubated without shaking. The test tubes containing the anaerobic cultures were capped with butyl rubber septa and polycarbonate screw caps, and purged with pure nitrogen gas. Optical density at 600 nm (OD_600_) was measured using a mini photo 518R photometer (Taitec, Tokyo, Japan). When necessary, 50 μg/ml kanamycin (Km) or 15 μg/ml gentamicin (Gm) was added to a culture medium. Agar plates contained 1.6% Bacto Agar (Difco).

**Table 1:** Bacterial strains and plasmids used in this study.

Strain or plasmid	Relevant characteristic	Source or reference
**Bacterial strains**		
*Escherichia coli*		
WM6026	Donor strain for conjugation; *lacI*^q^, *rrnB3*, DE*lacZ4787*, *hsdR514*, DE(*araBAD*)*567*, *E*(*rhaBAD*)*568*, *rph-1*, *att-lambda*::*pAE12-*del(*oriR6K-cat*::*frt5*), DE(*endA*)::*frt*, *uidA*(*delMluI*)::*pir*(wt), *attHK*::pJK1006-*del1/2* (del*oriR6K-cat*::*frt5*, del*trfA*::*frt*)	William Metcalf, University of Illinois
BL21 (DE3)	F^-^ *ompT hsdR17*(*r_B_^-^ m_B_^+^*) *gal dcm*(DE3) F^-^, *ompT*, *hsdS*_B_(r_B_^-^ m_B_^-^), *gal*(aaacI 857, *ind*1, *Sam*7, *nin*5, *lacUV*5-T7*gene*1), *dcm*(DE3)	Novagen
*Shewanella oneidensis*		
MR-1	Wild-type	ATCC (3)
Δ*crp*	The *crp* gene (SO_0624) disrupted	11
**Plasmids**		
pME*lacZ*	pME4510 derivative, *lacZ* Gm^r^	24
pME*lldP*+1	pME*lacZ* containing the region from +1 to +192 relative to TSP*_lldP_*	This study
pME*lldP*-60	pME*lacZ* containing the region from –60 to +192 relative to TSP*_lldP_*	This study
pME*lldP*-182	pME*lacZ* containing the region from –182 to +192 relative to TSP*_lldP_*	This study
pME*lldP*-541	pME*lacZ* containing the region from –541 to +192 relative to TSP*_lldP_*	This study
pBBR1MCS-2	Broad-host-range vector, *lacZ* promoter, Km^r^	22
pBBR1MCS-5	Broad-host-range vector, *lacZ* promoter, Gm^r^	22
pBBR*crp*	pBBR1MCS-5 containing *crp*	This study
pBBR*dld*	pBBR1MCS-2 containing *dld*	This study
pET-crp	pET-28(a)-based plasmid expressing *N-his-crp*	11

To construct plasmid pBBR*crp*, the *crp* gene was amplified using Phusion High-Fidelity DNA polymerase (New England Biolabs, Beverly, MA, United States) and primers crp-F-EcoRI and crp-R-BamHI (Supplementary Table S1). The PCR product was digested using EcoRI and BamHI, and cloned between the corresponding sites of pBBR1MCS-5 (Kovach et al., 1995). To construct plasmid pBBR*dld*, the *dld* gene was amplified using primers dld-F-BamHI and dld-R-XbaI (Supplementary Table S1). The PCR product was digested using BamHI and XbaI, and cloned between the corresponding sites of pBBR1MCS-2 (Kovach et al., 1995). The resulting plasmids, pBBR*crp* and pBBR*dld*, were introduced into Δ*crp* cells by filter mating with *E. coli* WM6026 (Kouzuma et al., 2010).

### Measurements of D- and L-Lactate

Concentrations of D- and L-lactate in culture supernatants were determined according to a method described previously (Nakagawa et al., 2015). *S. oneidensis* strains were cultivated in liquid MM until a stationary growth phase, and cells were removed by filtration through a membrane filter unit (0.20 μm pore size, DISMIC-13JP; Advantec, Tokyo, Japan). Concentrations of D- and L-lactate in the filtrate were measured using F-kit reagents (J. K. International, Tokyo, Japan) according to the manufacturer’s instructions.

### RNA Extraction

*Shewanella oneidensis* cells were cultivated in MM containing 10 mM racemic DL-lactate, D-lactate, or L-lactate under aerobic or 10 mM TMAO-reducing conditions and harvested at the logarithmic growth phase (OD_600_ of 0.2–0.3 or 0.04–0.06, respectively). RNA was extracted from the cells using a Trizol reagent (Invitrogen, Carlsbad, CA, United States) according to the manufacturer’s instructions. The extracted RNA was purified using an RNeasy Mini Kit and RNase-Free DNase Set (Qiagen, Valencia, CA, United States). The quality of purified RNA was evaluated using an Agilent 2100 Bioanalyzer with RNA 6000 Pico reagents and RNA Pico Chips (Agilent Technologies, Santa Clara, CA, USA) according to the manufacturer’s instructions.

### Reverse Transcription (RT)-PCR

To synthesize cDNA, 1.0 μg of total RNA extracted from MR-1 cells grown in MM containing 10 mM racemic DL-lactate as described above was reverse-transcribed using Superscript III Reverse Transcriptase (Invitrogen) and Random Primers (Invitrogen) according to the manufacturer’s instructions. The synthesized cDNA was amplified using Ex Taq DNA polymerase (Takara, Tokyo, Japan) and the primer sets listed in Supplementary Table S1. Amplification steps consisted of an initial denaturation step at 96°C for 30 s, followed by 30 amplification cycles of 96°C for 30 s, 52°C for 30 s, and 72°C for 2 min, and a final extension step at 72°C for 7 min. The PCR products were electrophoresed on 2% agarose gels. Negative control reactions without reverse transcriptase were also performed.

### Quantitative RT-PCR (qRT-PCR)

Quantitative RT-PCR (qRT-PCR) was performed using a LightCycler 1.5 instrument (Roche, Indianapolis, IN, United States) according to a method described previously (Kouzuma et al., 2014). A PCR reaction mixture contained 15 ng total RNA, 1.3 μL of 50 mM Mn(OAc)_2_ solution, 7.5 μL of LightCycler RNA Master SYBR Green I (Roche), and 0.15 μM primers listed in Supplementary Table S1. To generate standard curves, DNA fragments of target genes (*dld, lldP*, *lldF*, and 16S rRNA genes) were amplified by PCR using Ex Taq DNA polymerase (Takara) and the primer sets listed in Supplementary Table S1, and purified by gel electrophoresis using a QIAEX II Gel Extraction Kit (Qiagen) according to the manufacturer’s instructions. Standard curves were generated by amplifying a dilution series of the purified DNA fragments of each gene. The specificity of quantitative PCR was verified by a dissociation-curve analysis. Expression levels of target genes were normalized based on expression levels of the 16S rRNA gene.

### Identification of Transcriptional Start Sites

5′-Rapid amplification of cDNA ends (5′-RACE) PCR reactions were carried out using 1.0 μg total RNA extracted from MR-1 cells grown in 10 mM racemic DL-lactate up to the logarithmic growth phase and a SMATer RACE cDNA Amplification Kit (Takara) in accordance with the manufacturer’s instructions. The first single-strand cDNA was synthesized using the gene-specific primer lldP_race_out (Supplementary Table S1) and subsequently amplified using Universal Primer A Mix (Takara) and the primer lldP_race_in (Supplementary Table S1). The amplified DNA fragments were purified using a QIAquick PCR Purification Kit (Qiagen). The Purified DNA fragments were cloned into T-Vector pMD20 (Takara) and sequenced to determine the 5′-end points.

### LacZ Reporter Assay

To construct a series of reporter plasmids containing promoter regions upstream of *lldP*, these DNA regions were amplified using Phusion High-Fidelity DNA polymerase (New England Biolabs) and the primer sets listed in Supplementary Table S1. After purification using a QIAquick PCR Purification Kit (Qiagen), the DNA fragments were digested with EcoRI and SalI, and cloned between the corresponding sites of pME*lacZ* (Endoh et al., 2003). The constructed reporter plasmids were introduced into MR-1 and *Δcrp* by electroporation according to a method reported previously (Choi et al., 2006). The resulting reporter strains were aerobically cultivated in MM containing 10 mM racemic DL-lactate up to an OD_600_ of 0.3–0.5. β-Galactosidase activity was measured in triplicate according to the method of Miller (1972).

### EMSA

The electrophoretic mobility shift assay (EMSA) using purified N-terminal histidine-tagged CRP (N-his-CRP) was performed according to a method described previously (Kasai et al., 2015). Briefly, N-his-CRP was produced by *E. coli* BL21(DE3) harboring pET-crp (Kasai et al., 2015), and purified using a QuickPick IMAC Metal Affinity Kit for Proteins (Bio-Nobile, Turku, Finland). The purity of the protein samples was assessed by sodium dodecyl sulfate–polyacrylamide gel electrophoresis (SDS–PAGE) and Coomassie blue staining. Cy3-labeled DNA probes PB*lldP*1 and PB*lldP*2 were generated by PCR with the 5′-Cy3 labeled primer set listed in Supplementary Table S1. PB*lldP*3 and PB*lldP*3m were generated by annealing of the complementary single-strand oligonucleotides listed in Supplementary Table S1. DNA-binding reactions were performed in 20 μl of a previously described reaction mixture (Kasai et al., 2015) containing 2 nM Cy3-labeled DNA probe, 50 ng N-his-CRP, and 50 μM cAMP.

## Results

### Deficient D-Lactate Utilization by *Δcrp*

**FIGURE 1:**
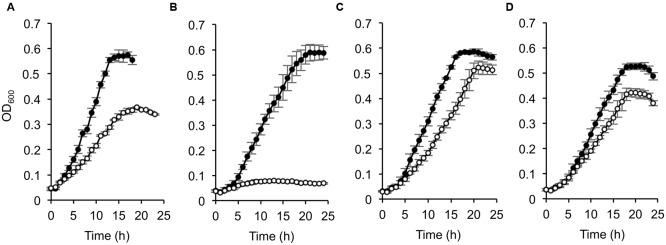
**Growth of wild-type (WT) and Δ*crp* on DL-lactate (A)**, D-lactate **(B)**, L-lactate **(C)**, and pyruvate **(D)**. WT (closed circle) and Δ*crp* (open circle) were aerobically grown in minimal medium (MM) containing 10 mM of each substrate. Error bars represent standard deviations calculated from at least triplicate independent cultures.

**FIGURE 2:**
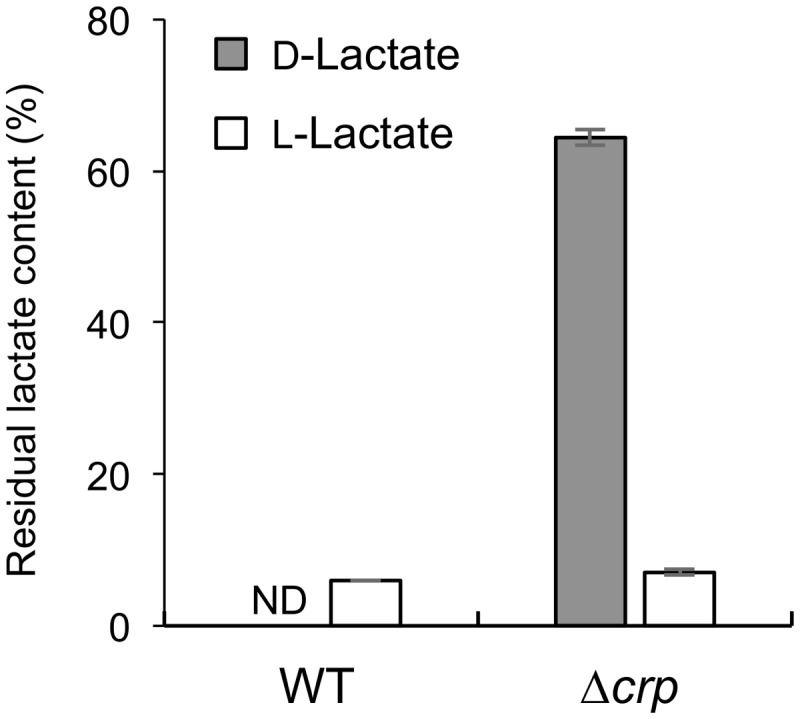
**Residual D- and L-lactate contents in WT and Δ*crp* cultures.** Error bars represent standard deviations calculated from at least triplicate independent experiments. ND, not detected.

To investigate the involvement of CRP in the catabolism of D-lactate and other organic acids in *S. oneidensis* MR-1, we compared growth characteristics of wild-type MR-1 (WT) and *Δcrp* (Kasai et al., 2015) on a 10 mM racemic mixture of D- and L-lactate (DL-lactate; 5 mM each isomer), D-lactate, L-lactate, and pyruvate (**Figure 1**). When these strains were grown on DL-lactate, the final cell density of *Δcrp* was approximately half that of WT (**Figure 1A**). Measurements of residual D- and L-lactate in the culture media (**Figure 2**) revealed that a large portion of D-lactate remained in the *Δcrp* culture. These results suggest that *Δcrp* has a decreased capacity for metabolizing D-lactate. This notion is supported by the growth deficiency of this mutant in the medium containing D-lactate as the sole substrate (**Figure 1B**). Complementary expression of *crp* in Δ*crp* restored the ability to grow on D-lactate (Supplementary Figure S1), demonstrating the involvement of CRP in D-lactate utilization. However, *Δcrp* was capable of growing on L-lactate and pyruvate, albeit that growth rates and final cell densities were slightly lower than those of WT (**Figures 1C,D**). These results suggest that CRP is required for the expression of gene(s) involved in D-lactate utilization, such as the *dld* gene encoding D-LDH in MR-1, although this regulator is not essential for the expression of genes involved in L-lactate and pyruvate utilization.

### Involvement of CRP in the Expression of *dld*

**FIGURE 3:**
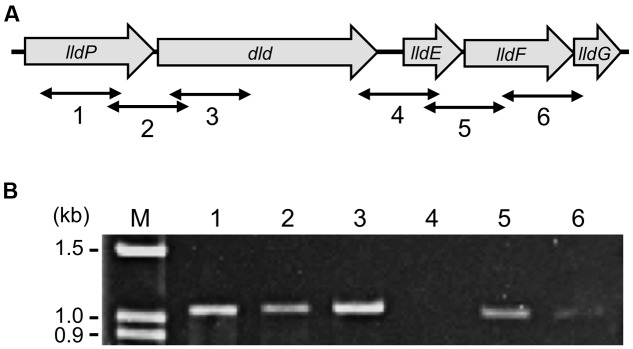
**Organization and transcriptional units of the *dld* and *lld* genes. (A)** Schematic illustration of the organization of the *dld* and *lld* genes. Bidirectional arrows indicate target regions of the RT-PCR analysis. **(B)** RT-PCR of the *dld* and *lld* genes. RNA was extracted from WT cells grown aerobically in MM containing 10 mM DL-lactate until the logarithmic growth phase. Lane numbers correspond to the target regions shown in **A**. Left-side numbers indicate molecular sizes (kb) of bands in the marker (lane M).

In the MR-1 genome, the *dld* gene is located within a gene cluster consisting of *lldP* (a putative lactate permease gene)*, dld*, and *lldEFG* (SO_1522 to SO_1518; **Figure 3A**) (Pinchuk et al., 2009). Thus, to examine the involvement of CRP in the transcription of genes in this cluster, we first determined transcriptional units by RT-PCR and subsequently compared amounts of transcripts from these genes in WT and Δ*crp* by qRT-PCR. RT-PCR analysis using total RNA extracted from WT cells grown on DL-lactate detected a transcript containing the intergenic region between *lldP* and *dld* (**Figure 3B**, lane 2), indicating the polycistronic transcription of these two genes. The analysis also amplified the *lldE*–*lldF* and *lldF*–*lldG* intergenic regions (**Figure 3B**, lanes 5 and 6). However, no transcripts were detected when the *dld*–*lldE* intergenic region was analyzed (**Figure 3B**, lane 4), demonstrating that the transcription of *dld* is terminated within this region. These results indicate that the five genes in this gene cluster are transcribed as two operons, the *lldP-dld* and *lldEFG* operons.

**FIGURE 4:**
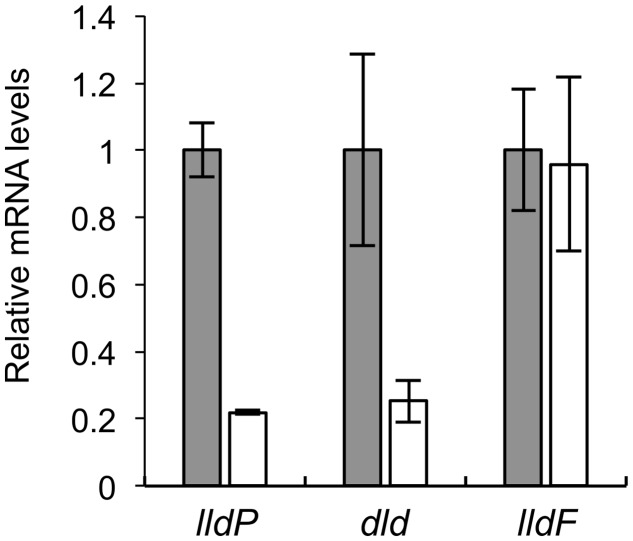
**Quantitative RT-PCR (qRT-PCR) analyses of *lldP*, *dld*, and *lldF* in WT (gray bars) and *Δcrp* (white bars).** Cells were aerobically grown in MM containing 10 mM DL-lactate and harvested in the logarithmic growth phase. Results are expressed as relative values to mRNA levels in the WT cells. Error bars represent standard deviations calculated from at least three independent experiments.

We performed qRT-PCR analyses to determine expression levels of *lldP*, *dld*, and *lldF* in WT and *Δcrp* (**Figure 4**). The results revealed that expression levels of *lldP* and *dld* in *Δcrp* were decreased to approximately 20% of those in WT, although the expression of *lldF* did not significantly differ between these two strains. This result indicates that CRP is required for the transcriptional activation of the *lldP-dld* operon, although this regulator is not involved in the transcription of the *lldEFG* operon. Decreased expression of *lldE* and *dldP* in *Δcrp* was also observed when cells were grown under TMAO-reducing conditions (Supplementary Figure S2), indicating that CRP activates the transcription of these genes under both aerobic and anaerobic conditions.

### Restored D-Lactate Utilization by *dld*-Complemented Δ*crp*

**FIGURE 5:**
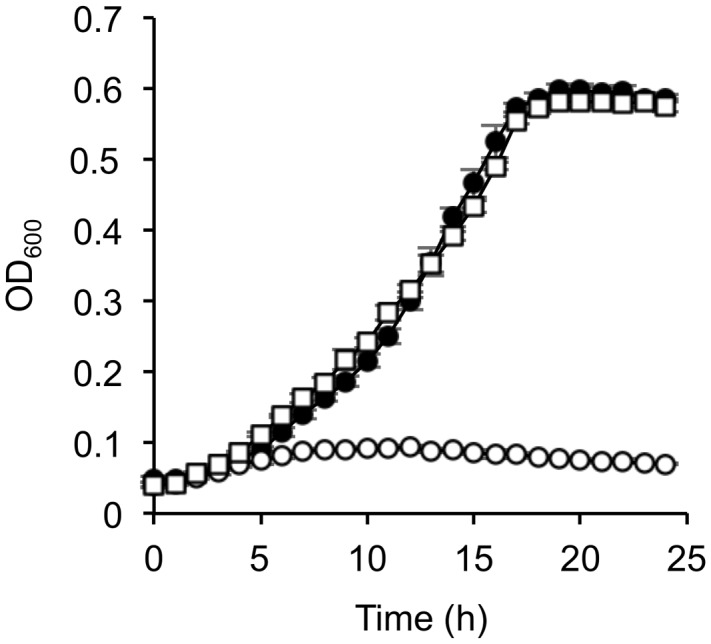
**Growth of *dld-*complemented *Δcrp* on D-lactate.** WT harboring the control vector pBBR1MCS-2 (closed circle), *Δcrp* harboring pBBR1MCS-2 (open circle), and *Δcrp* harboring pBBR*dld* (open squire) were aerobically grown in MM containing 10 mM D-lactate. Error bars represent standard deviations calculated from at least three independent experiments.

The above-mentioned results suggest that the decreased expression of the *lldP*-*dld* operon in Δ*crp* is related to the deficient growth of this mutant on D-lactate. However, it remained possible that transcriptional activation of *lldP* and/or other CRP-regulated genes might also be required for D-lactate utilization. To test this possibility, we transformed Δ*crp* with a plasmid constitutively expressing the *dld* gene, pBBR*dld* (**Table 1**), and examined the growth of the resultant strain, Δ*crp*(pBBR*dld*), on D-lactate. When cells were grown under aerobic conditions (**Figure 5**), the growth of Δ*crp*(pBBR*dld*) on D-lactate was comparable to that of the control strain, MR-1(pBBR1MCS-2). The restored growth of Δ*crp*(pBBR*dld*) on D-lactate was also observed when cells were grown under TMAO-reducing conditions (Supplementary Figure S3), although its growth was slower than that of MR-1(pBBR1MCS-2). We consider that this growth retardation is due to the decreased ability of Δ*crp* to grow under anaerobic conditions because similar growth retardation was observed when Δ*crp*(pBBR1MCS-2) was cultivated using L-lactate and TMAO (Supplementary Figure S4). We therefore concluded that the deficient growth of Δ*crp* on D-lactate is attributable to the decreased expression of the *dld* gene.

### D-Lactate-Independent Expression of the *lldP-dld* Operon

**FIGURE 6:**
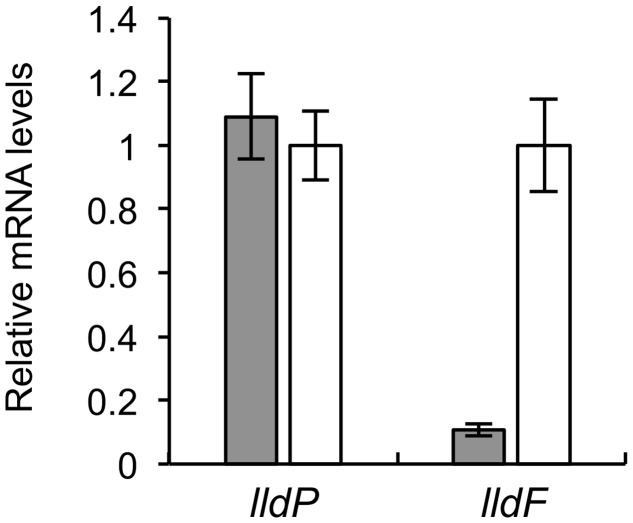
**Quantitative RT-PCR analyses of *lldP* and *lldF* in WT grown on D-lactate (gray bars) or L-lactate (white bars).** Cells were grown in MM containing 10 mM D- or L-lactate and harvested in the logarithmic growth phase. Data are expressed as relative values to mRNA levels in cells grown on L-lactate. Error bars represent standard deviations calculated from at least three independent experiments.

A previous study reported that D-LDH activity in MR-1 was independent of growth substrates, whereas L-LDH activity was increased when it was grown on L-lactate (Brutinel and Gralnick, 2012). However, it was unclear how the presence of D- and L-lactate affects transcription of the *lldP-dld* and *lldEFG* operons. To address this, we cultivated MR-1 cells in the presence of D- or L-lactate, and investigated the expression levels of *lldP* and *lldF* by qRT-PCR analyses (**Figure 6**). We found that, when cells were grown on L-lactate, the expression of *lldF* was markedly increased, suggesting that the *lldEFG* operon is regulated by an L-lactate-dependent regulatory mechanism (i.e., the LlpR transcriptional regulator) (Brutinel and Gralnick, 2012). In contrast, the expression of *lldP* did not significantly differ between the two growth conditions. These results support the idea that the *lldP-dld* and *lldEFG* operons are regulated by different mechanisms, and that expression of the *lldP-dld* operon is not affected by the presence or absence of D-lactate.

### Identification of the *lldP* Promoter Region

**FIGURE 7:**
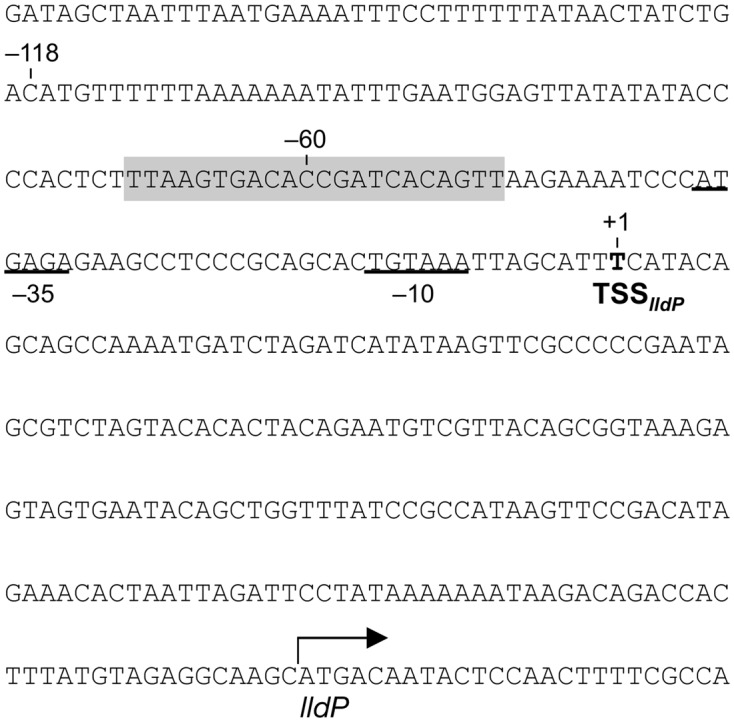
**Nucleotide sequence upstream of *lldP*.** The position of TSS*_lldP_* is indicated by boldface. Nucleotides are numbered relative to TSS*_lldP_* (+1). Putative –10 and –35 promoter sequences are underlined. A shaded sequence indicates a putative CRP-binding motif.

To investigate the transcriptional mechanism of the *lldP*-*dld* operon in more detail, we determined a transcription start site (TSS) and upstream promoter region for the *lldP*-*dld* operon. 5′-RACE PCR analysis detected a TSS 192 bp upstream of the ATG start codon of *lldP* (TSS*_lldP_*; **Figure 7**). A sequence similar to the consensus sequence of the *E. coli* σ^70^-dependent promoter (–10 and –35 regions) (deHaseth et al., 1998) was found in the upstream region of TSS*_lldP_* (**Figure 7**). Although a previous study reported that the RpoE-binding motif is detected upstream of many anaerobic respiratory genes in MR-1 (Barchinger et al., 2016), a sequence similar to that motif was not found in the upstream of *lldP*. However, a candidate CRP-binding sequence (5′-TTAAGTGACACCGATCACAGTT-3′) predicted by Gao et al. (2010) was located in position –70 to –49 relative to TSS*_lldP_*. We therefore hypothesized that CRP binds to this sequence and activates the downstream *lldP* promoter (P*_lldP_*).

**FIGURE 8:**
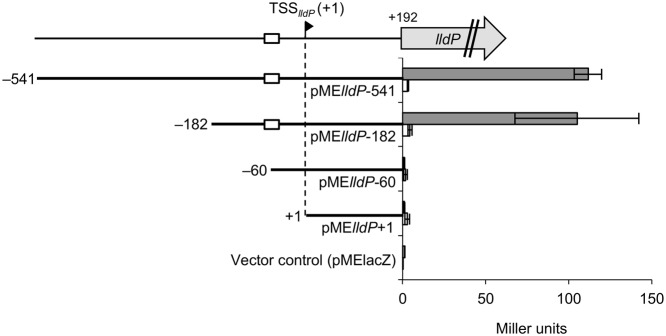
**5′-deletion analyses of the upstream region of *lldP*.** WT (gray bars) or Δ*crp* (white bars) cells harboring the pME-series plasmids were grown on DL-lactate, and their LacZ activities were measured in the logarithmic growth phase. White boxes represent the putative CRP-binding motif. Error bars show standard deviations calculated from at least three independent experiments.

To determine the DNA regions involved in the activation of P*_lldP_* by CRP, we performed 5′-deletion analysis of the sequence upstream of TSS*_lldP_*. For this purpose, WT and Δ*crp* strains were transformed with the *lacZ* reporter plasmids with promoter region deletions (pME*lldP*-541 to pME*lldP*+1; **Table 1**), and resultant transformants were grown on DL-lactate until the logarithmic growth phase. Measurement of the LacZ activities of these strains (**Figure 8**) revealed that, while Δ*crp* cells transformed with these reporter plasmids consistently exhibited very low levels of LacZ activities, WT cells transformed with pME*lldP*-541 and pME*lld*-182 exhibited much higher LacZ activities than these Δ*crp* cells. This result supports the idea that CRP plays a critical role in the activation of P*_lldP_*. The analysis also revealed that LacZ activity in WT cells was decreased to the same level as that in Δ*crp* cells when they were transformed with pME*lld*-60. This result demonstrates that the activation of P*_lldP_* by CRP requires the DNA region from –182 to –60 relative to TSS*_lldP_*, which partially contains the putative CRP-binding site (**Figure 7**).

### Binding of CRP to the *lldP* Promoter Region

**FIGURE 9:**
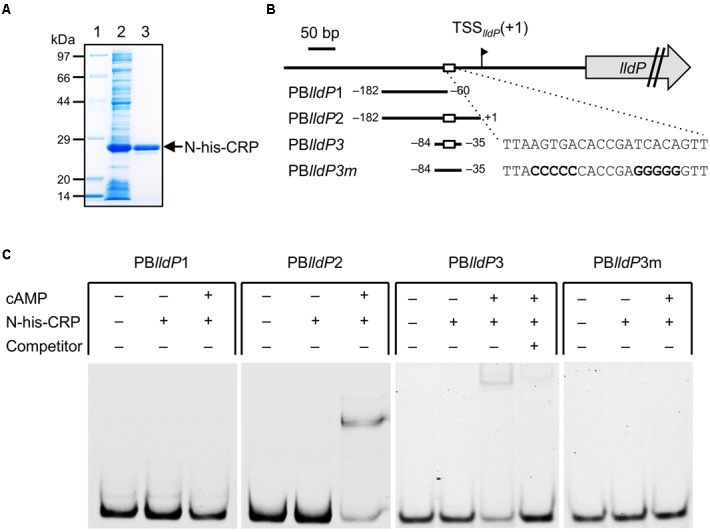
**Electrophoretic mobility shift assay (EMSA) using CRP and upstream regions of *lldP*. (A)** Sodium dodecyl sulfate–polyacrylamide gel electrophoresis (SDS–PAGE) of N-his-CRP protein samples. Protein samples (5 μg) were analyzed on 12.5% SDS-polyacrylamide gels. Lane 1, *Escherichia coli* BL21 (DE3) (pET-crp) crude extract; lane 2, purified N-his-CRP; lane 3, molecular weight marker. **(B)** DNA fragments used as probes. Positions of 5′ and 3′ ends of the fragments relative to TSS*_lldP_* (+1) are shown. White boxes represent the putative CRP-binding motif. The mutated sequences in PB*lldP*3m are shown in bold. **(C)** Binding of CRP to each probe. DNA-binding reactions were performed in the presence (+) or absence (–) of CRP, cAMP, and specific competitor (2 μM unlabeled PB*lldP*3 probe).

To investigate whether CRP binds directly to the upstream activation region of P*_lldP_*, we performed an EMSA using purified CRP protein (**Figure 9A**) and DNA probes containing the upstream regions of P*_lldP_* (**Figure 9B**). When a DNA probe containing the region from –182 to –61 relative to TSS*_lldP_* (PB*lld*P1) was mixed with CRP, no shifted bands were observed (**Figure 9C**). However, when a probe containing the region from –182 to –1 relative to TSS*_lldP_* (PB*lld*P2) was used, a shifted band was detected in a cAMP-dependent manner (**Figure 9C**), indicating that CRP directly binds to this region. Together with the results of the 5′-deletion analysis, these results suggested that CRP binds to a region around position –60, which is consistent with the position of the putative CRP-binding motif (**Figure 7**). To examine the binding of CRP to this motif, we performed an EMSA using a 50-bp probe containing the region from –84 to –35 (PB*lldP*3) and a mutated probe in which the core motif sequence was replaced (PB*lldP*3m). The results revealed that CRP specifically bound to PB*lldP*3, but not to PB*lld*3m (**Figure 9C**). We therefore conclude that this motif is involved in the binding of CRP and the transcriptional activation of the *lldP*-*dld* operon.

## Discussion

Like many other anaerobic respiratory bacteria, *Shewanella* spp. preferentially catabolize low-molecular-weight organic acids (Serres and Riley, 2006; Fredrickson et al., 2008), and studies have used lactate (mostly racemic DL-lactate) as a substrate for cultivating *S. oneidensis* MR-1. Despite the importance of lactate as a catabolic substrate for *Shewanella* spp., however, little is known about how these bacteria regulate catabolic pathways for this compound. Here, we demonstrated that CRP plays a critical role in the transcriptional regulation of the *dld* gene encoding D-LDH in MR-1. As CRP is also involved in the expression of many anaerobic respiratory genes (Saffarini et al., 2003; Kouzuma et al., 2015), we suggest that MR-1 uses this transcriptional regulator to coordinately regulate D-lactate metabolism and anaerobic respiration. A previous study reported that, in *Corynebacterium glutamicum*, a CRP/Fnr-type global transcriptional regulator, GlxR, binds to promoter regions of the *ldhA* gene encoding a fermentative L-LDH and the *narKGHJI* operon encoding nitrate respiratory enzymes in a cAMP-dependent manner (Kohl et al., 2008). It is therefore likely that a broad range of bacteria utilizes cAMP-dependent regulatory mechanisms for the coordinated expression of catabolic and respiratory pathways.

In many bacteria, lactate is oxidized to pyruvate by NADH-independent LDHs (iLDHs) (Garvie, 1980; Jiang et al., 2014). For example, in *E.* *coli* and *C. glutamicum*, D-lactate is catabolized through membrane-bound D-iLDHs that utilize membrane-associated quinones as electron-accepting cofactors (Dym et al., 2000; Kato et al., 2010). *S. oneidensis* MR-1 also oxidizes D-lactate by D-iLDH (Dld) (Pinchuk et al., 2009), and is thereby likely to reduce membrane quinones. In this strain, electrons accumulated in the membrane quinone pool are transferred to anaerobic electron acceptors, such as fumarate, DMSO, and metal oxides, via an inner membrane-anchored cytochrome, CymA, and periplasm- and outer membrane-localized proteins, such as FccA, DmsABEF, MtrCAB, and OmcA (Beliaev et al., 2001; Myers and Myers, 2002; Gralnick et al., 2006; Shi et al., 2006; Hartshorne et al., 2009; Schuetz et al., 2009; Fonseca et al., 2012). It is therefore suggested that D-lactate oxidation in MR-1 is metabolically linked to anaerobic respiratory pathways via membrane-associated quinones.

D- and L-Lactate are major fermentation end products from glucose and other carbohydrates. Many fermentative microbes, including lactic acid bacteria, produce these organic acids and release them into the environment (Garvie, 1980). It is therefore possible that *Shewanella* spp. utilize these fermentation products as catabolic substrates in their habitats. Here, we demonstrate that the catabolic pathways of D- and L-lactate in MR-1 are regulated by different mechanisms. Expression analysis in the presence of D- or L-lactate (**Figure 6**) revealed that expression of the *lldEFG* operon (encoding L-LDH) is induced by L-lactate, while expression of the *lldP*-*dld* operon (encoding D-LDH) is independent of the presence of D- or L-lactate. Considering that the *lldP*-*dld* operon is regulated by CRP, this operon is likely regulated in an intracellular energy status-dependent manner. It is therefore interesting to consider why only D-lactate catabolism is regulated in a substrate-independent manner. We speculate that this difference reflects how these lactate isomers are supplied to MR-1 in its habitats. The substrate-dependent regulation of the L-LDH genes suggests that the supply of L-lactate may be a relatively rare event in such environments, and thus MR-1 expresses these genes only when L-lactate is available. In contrast, the CRP-dependent regulation of the *dld* gene implies that MR-1 is frequently exposed to D-lactate in its habitats (mostly anaerobic environments). This notion is likely, given that MR-1 itself produces D-lactate as an electron sink when it catabolizes carbohydrates under electron acceptor-limited conditions. In addition to the respiratory D- and L-iLDH genes, MR-1 possesses the *ldhA* gene, which encodes a putative NADH-dependent D-LDH that catalyzes the fermentative production of D-lactate from pyruvate (Heidelberg et al., 2002), suggesting that this strain has the ability to produce D-lactate under electron acceptor-limited conditions. It is likely that MR-1 produces D-lactate from carbohydrates by LdhA as a temporal electron sink when electron acceptors are limited, and subsequently catabolizes D-lactate using Dld and anaerobic respiratory chains. Supporting this speculation, we recently found that an engineered *S. oneidensis* strain capable of utilizing glucose produces D-lactate from this sugar when grown under electron acceptor (fumarate)-limited conditions (Nakagawa et al., 2015). Gene-knockout experiments also demonstrate that LdhA and Dld catalyze the production and degradation of D-lactate, respectively, under these conditions (Nakagawa et al., 2015). The wild-type MR-1 cannot utilize glucose but has the ability to utilize other carbohydrates, such as *N*-acetylglucosamine (Yang et al., 2006; Rodionov et al., 2010). We therefore speculate that these carbohydrates are important catabolic substrates for *Shewanella* spp., and that D-lactate metabolism plays a role in maintaining the intracellular redox balance. Interestingly, a previous study also reported that a putative CRP-binding site is present upstream of *ldhA*, and that expression of the *ldhA* gene is up-regulated under oxygen-limited conditions, as is found for *dld* and anaerobic respiratory genes (Barchinger et al., 2016). We therefore hypothesize that the cAMP/CRP system contributes more widely to the regulation of catabolic pathways in *Shewanella* than previously thought.

The present study also demonstrates that the *dld* gene is co-transcribed with the *lldP* gene encoding a putative lactate permease (**Figures 3A,B**). In *E. coli*, the L-lactate permease LldP and glycolate permease GlcA are both involved in the uptake of D-lactate, although no specific D-lactate permeases are found (Núñez et al., 2002). LldP in MR-1 shows low but significant homologies to LldP and GlcA in *E. coli* (22.8 and 24.8% identities, respectively, in amino acid sequences), suggesting the involvement of this protein in the uptake of D- and/or L-lactate. In addition to *lldP*, the MR-1 genome encodes another putative lactate permease, LctP (SO_0827), which shows high homologies to LldP and GlcA in *E. coli* (66.0 and 65.7% identities, respectively). It is therefore likely that MR-1 takes up D- and/or L-lactate using one or both of these permeases. Notably, a putative CRP-binding site is also found in the upstream region of the *lctP* gene (Barchinger et al., 2016), suggesting that expression of the *lldP* and *lctP* genes is controlled by a common transcriptional regulator. It would therefore be interesting to investigate the presence of functional differences between *lldP* and *lctP*. In this regard, we showed that the growth deficiency of Δ*crp* on D-lactate was restored by introducing the plasmid from which only Dld was expressed (**Figure 5**), indicating that transcriptional activation of *lldP* by CRP is not essential for growth on D-lactate. This result suggests two possibilities for D-lactate uptake in MR-1: (i) LldP expressed at a low level is sufficient for this strain to grow on D-lactate, and (ii) uptake is mainly due to the alternative D-lactate permease, LctP. Confirming these hypotheses requires additional investigation, such as knockout analyses of the permease genes.

While the present study clearly demonstrates that CRP is involved in transcriptional regulation of the *lldP*-*dld* operon, further studies are needed to fully understand the regulatory systems for this operon. For instance, although the intracellular cAMP concentration is a key determinant for the expression of CRP regulons, including the *lldP*-*dld* operon, it is currently unclear how the concentration of this signaling molecule is controlled in *Shewanella*. Since previous studies have shown that many CRP-regulated genes are up-regulated under electron acceptor-limited conditions (Wang et al., 2012; Kasai et al., 2015; Barchinger et al., 2016), cAMP concentration may be affected by cellular redox and/or energy states. In addition, it has been previously found that expression of the *dld* gene is up-regulated in the presence of a high-potential electrode in a bioelectrochemical reactor, while that of the *ldhA* gene is not affected by the electrode potential (Nakagawa et al., 2015). This result implies that an additional regulatory factor(s) is involved in the expression of the *lldP*-*dld* operon and/or the *ldhA* gene. We expect that further investigation of this operon will reveal as yet unexplored regulatory mechanisms in bacteria.

## Author Contributions

TK carried out the majority of the experimental work and drafted the manuscript. AK conceived of the study, participated in its design and coordination, and drafted the manuscript. KW supervised the study and performed manuscript editing. All authors read and approved the final manuscript.

## Conflict of Interest Statement

The authors declare that the research was conducted in the absence of any commercial or financial relationships that could be construed as a potential conflict of interest.
