# Supplementary material for: CRP Regulates D-Lactate Oxidation in Shewanella oneidensis MR-1
Source: Front Microbiol. 2017 May 16;8:869. doi: 10.3389/fmicb.2017.00869 (PMC5432575; doi:10.3389/fmicb.2017.00869)
Supplement: Supplementary file 1 [file Presentation_1.PDF]

# CRP regulates D-lactate oxidation in *Shewanella oneidensis* MR-1

Takuya Kasai, Atsushi Kouzuma*, and Kazuya Watanabe

School of Life Sciences, Tokyo University of Pharmacy and Life Sciences, Hachioji, Tokyo, Japan

*Corresponding author
E-mail: akouzuma@toyaku.ac.jp

**Supplemental Materials:**

**Table S1.** Primers used in this study.

**Figure S1.** Growth of *crp*-complemented Δ*crp* on D-lactate.

**Figure S2.** qRT-PCR analyses of *lldP* and *dld* in WT and Δ*crp* grown under TMAO-reducing conditions.

**Figure S3.** Growth of *dld*-complemented Δ*crp* on D-lactate under TMAO-reducing conditions.

**Figure S4.** Growth of Δ*crp*(pBBR1MCS-2) on L-lactate under TMAO-reducing conditions.

**Table S1. Primers used in this study.**

| Primer | Sequence (5′–3′) | **Modification**, for use |
|---|---|---|
| qRT-16S-F | AGCGCAACCCCTATCCTTAT | qRT-PCR for 16S rRNA gene |
| qRT-16S-R | CGTAAGGGCCATGATGACTT | qRT-PCR for 16S rRNA gene |
| qRT-dld-II-F | TGATGCTCAAACGCTCGAAC | qRT-PCR for *dld-II* gene |
| qRT-dld-II-R | AAAAGCCCTTTACGCATCGC | qRT-PCR for *dld-II* gene |
| qRT-lldP-F | GGCGTTTCATGCAAGTC | qRT-PCR for *lldP* gene |
| qRT-lldP-R | ATCGACGCAAGACCTGC | qRT-PCR for *lldP* gene |
| RT-lldP-F | CGCCGCCGTGTTTATTTGGC | RT-PCR, region 1 |
| RT-lldP-R | GGATTGCTTAATCGCAGGGC | RT-PCR, region 1 |
| RT-lldP-dld-II-F | CGCCACTCAAAGCTTGGC | RT-PCR, region 2 |
| RT-lldP-dld-II-R | CCAATAACGGCCGCGCC | RT-PCR, region 2 |
| RT-dld-II-F | CCGTTTTGCTTGGTCTACCG | RT-PCR, region 3 |
| RT-dld-II-R | GCCCTTTACGCATCGCCC | RT-PCR, region 3 |
| RT-dld-II-lldE-F | GAGCCCAAGATGCAAGCG | RT-PCR, region 4 |
| RT-dld-II-lldE-R | CGGCTCCTTCATCGACGGC | RT-PCR, region 4 |
| RT-lldEF-F | CAGCCTTGCTACGCCAAGCG | RT-PCR, region 5 |
| RT-lldEF-R | GGTTAGCGCCCATATCGGC | RT-PCR, region 5 |
| RT-lldFG-F | CTAAGGCTGGCGAGTCCG | RT-PCR, region 6 |
| RT-lldFG-R | GCAGTGCCGCAAGGCCGC | RT-PCR, region 6 |
| lldP_race_in | ACAACGGATGCGGCCAATAGAGTGG | 5′ RACE PCR |
| lldP_race_out | GCGCCGAAGATGATGGTTAAGGGGG | 5′ RACE PCR |
| lldP_+192-R | GATC**GTCGAC**GCTTGCCTCTACATAAAGTGG | **SalI**, LacZ reporter assay |
| lldP_+1-F | GATC**GAATTC**TCATACAGCAGCCAAAATG | **EcoRI**, LacZ reporter assay |
| lldP_-118-F | GATC**GAATTC**CATGTTTTTTAAAAAAATATTTG | **EcoRI**, LacZ reporter assay |
| lldP_-182-F | GATC**GAATTC**GGATCAGTTAATTGCAATAC | **EcoRI**, LacZ reporter assay |
| lldP_-360-F | GATC**GAATTC**CAAAGCGTGACCAGAATCAC | **EcoRI**, LacZ reporter assay |
| lldP_-541-F | GATC**GAATTC**GCCGTATCGCAGCAAAAGCC | **EcoRI**, LacZ reporter assay |
| lldP_EMSA-182_F | GGATCAGTTAATTGCAATAC | 5′-Cy3, PB*lldP*1, PB*lldP*2, EMSA |
| lldP_EMSA-61_R | TGTCACTTAAAGAGTGGGG | PB*lldP*1, EMSA |
| lldP_EMSA-1_R | AATGCTAATTTACAGTGCTG | PB*lldP*2, EMSA |
| lldP_EMSA-84-35_F | ATATACCCCACTCTTTAAGTGACACCGATCACAGTTA AGAAAATCCCATG | 5′-Cy3, PB*lldP*3, EMSA |
| lldP_EMSA-84-35_R | CATGGGATTTTCTTAACTGTGATCGGTGTCACTTAAA GAGTGGGGTATAT | PB*lldP*3, EMSA |
| lldP_EMSA-84-35m_F | ATATACCCCACTCTTTACCCCCCACCGAGGGGGGTT AAGAAAATCCCATG | 5′-Cy3, PB*lldP*3m, EMSA |
| lldP_EMSA-84-35m_R | CATGGGATTTTCTTAACCCCCCTCGGTGGGGGGTAA AGAGTGGGGTATAT | PB*lldP*3m, EMSA |
| dld-BamHI-F | CGC**GGATCC**ACCATCTTGTTCGAAGTCAC | **BamHI**, pBBR*dld* construction |
| dld-XbaI-R | CTAG**TCTAGA**CTTCTAAAGAAAAACGGGGC | **XbaI**, pBBR*dld* construction |
| crp_F_EcoRI | GGGG**GAATTC**TTTCGTTTTAATCAATCGAGG | **EcoRI**, pBBR*crp* construction |
| crp_R_BamHI | GGGG**GGATCC**GATACAGGCTTAAATCAAGCTG | **BamHI**, pBBR*crp* construction |

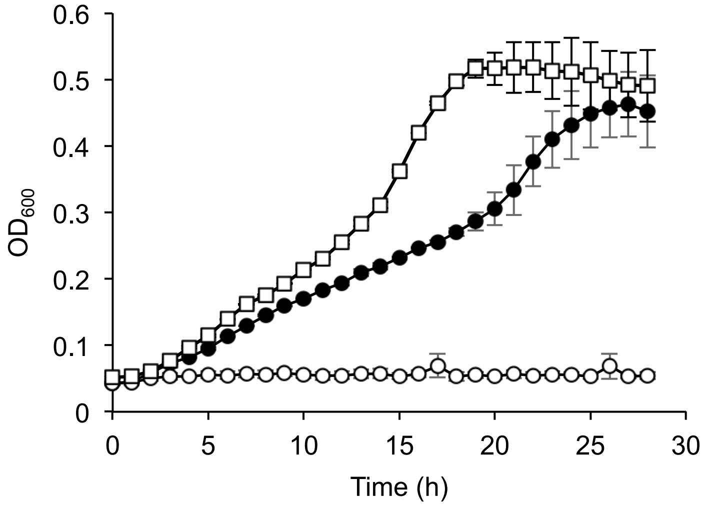


**Figure S1.** Growth of *crp*-complemented Δ*crp* on D-lactate. WT harboring the control vector pBBR1MCS-5 (closed circle), Δ*crp* harboring pBBR1MCS-5 (open circle), and Δ*crp* harboring pBBR*crp* (open squire) were aerobically grown in MM containing 10 mM D-lactate. Error bars represent standard deviations calculated from at least three independent experiments.

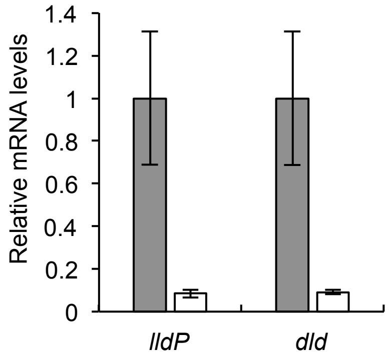


**Figure S2.** qRT-PCR analyses of *lldP* and *dld* in WT (gray bars) and Δ*crp* (white bars) grown under TMAO-reducing conditions. Cells were anaerobically grown in MM containing 10 mM DL-lactate and 10 mM TMAO, and harvested in the logarithmic growth phase. Results are expressed as relative values to mRNA levels in the WT cells. Error bars represent standard deviations calculated from at least three independent experiments.

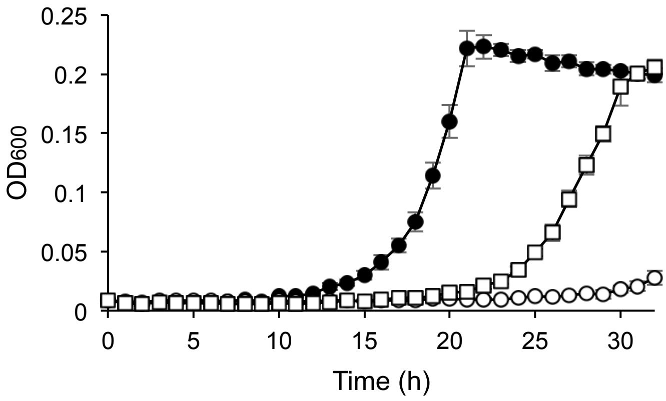


**Figure S3.** Growth of *dld*-complemented Δ*crp* on D-lactate under TMAO-reducing conditions. WT harboring the control vector pBBR1MCS-2 (closed circle), Δ*crp* harboring pBBR1MCS-2 (open circle), and Δ*crp* harboring pBBR*dld* (open squire) were anaerobically grown in MM containing 30 mM D-lactate and 30 mM TMAO. Error bars represent standard deviations calculated from at least three independent experiments.

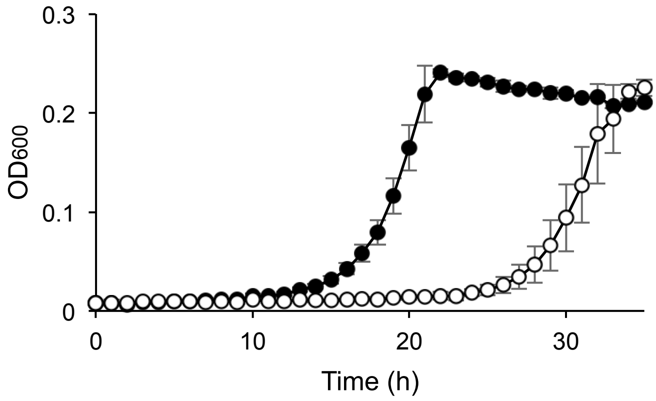


**Figure S4.** Growth of Δ*crp*(pBBR1MCS-2) on L-lactate under TMAO-reducing conditions. WT harboring pBBR1MCS-2 (closed circle) and Δ*crp* harboring pBBR1MCS-2 (open circle) were anaerobically grown in MM containing 30 mM L-lactate and 30 mM TMAO. Error bars represent standard deviations calculated from at least three independent experiments.
